# Development of an Instructional Design Evaluation Survey for Postgraduate Medical E-Learning: Content Validation Study

**DOI:** 10.2196/13921

**Published:** 2019-08-09

**Authors:** Robert Adrianus de Leeuw, Michiel Westerman, Kieran Walsh, Fedde Scheele

**Affiliations:** 1 Athena Institute for Trans-Disciplinary Research VU University Amsterdam Netherlands; 2 Amsterdam University Medical Center Amsterdam Netherlands; 3 Department of Internal Medicine Franciscus Gasthuis en Vlietland Hospital Rotterdam Netherlands; 4 British Medical Journal Learning British Medical Association House London United Kingdom

**Keywords:** postgraduate medical education, continuing medical education, e-learning, distance education, survey, evaluation

## Abstract

**Background:**

E-Learning has taken a firm place in postgraduate medical education. Whereas 10 years ago it was promising, it now has a definite niche and is clearly here to stay. However, evaluating the effect of postgraduate medical e-learning (PGMeL) and improving upon it can be complicated. While the learning aims of e-learning are evaluated, there are no instruments to evaluate the instructional design of PGMeL. Such an evaluation instrument may be developed by following the Association for Medical Education in Europe (AMEE) 7-step process. The first 5 steps of this process were previously performed by literature reviews, focus group discussion, and an international Delphi study.

**Objective:**

This study will continue with steps 6 and 7 and answer the research question: Is a content-validated PGMeL evaluation survey useful, understandable, and of added value for creators of e-learning?

**Methods:**

There are five phases in this study: creating a survey from 37 items (phase A); testing readability and question interpretation (phase B); adjusting, rewriting, and translating surveys (phase C); gathering completed surveys from three PGMeL modules (phase D); and holding focus group discussions with the e-learning authors (phase E). Phase E was carried out by presenting the results of the evaluations from phase D, followed by a group discussion. There are four groups of participants in this study. Groups A and B are experienced end users of PGMeL and participated in phase B. Group C are users who undertook e-learning and were asked to complete the survey in phase D. Group D are the authors of the e-learning modules described above.

**Results:**

From a list of 36 items, we developed a postgraduate Medical E-Learning Evaluation Survey (MEES). Seven residents participated in the phase B group discussion: 4 items were interpreted differently, 3 were not readable, and 2 items were double. The items from phase B were rewritten and, after adjustment, understood correctly. The MEES was translated into Dutch and again pilot-tested. All items were clear and were understood correctly. The MEES version used for the evaluation contained 3 positive domains (motivation, learning enhancers, and real-world translation) and 2 negative domains (barriers and learning discouragers), with 36 items in those domains, 5 Likert scale questions of 1 to 10, and 5 open questions asking participants to give their own comments in each domain. Three e-learning modules were evaluated from July to November 2018. There were a total of 158 responses from a Dutch module, a European OB/GYN (obstetrics and gynecology) module, and a surgical module offered worldwide. Finally, 3 focus group discussions took place with a total of 10 participants. Usefulness was much appreciated, understandability was good, and added value was high. Four items needed additional explanation by the authors, and a Creators’ Manual was written at their request.

**Conclusions:**

The MEES is the first survey to evaluate the instructional design of PGMeL and was constructed following all 7 steps of the AMEE. This study completes the design of the survey and shows its usefulness and added value to the authors. It finishes with a final, publicly available survey that includes a Creators’ Manual. We briefly discuss the number of responses needed and conclude that more is better; in the end, however, one has to work with what is available. The next steps would be to see whether improvement can be measured by using the MEES and continue to work on the end understandability in different languages and cultural groups.

## Introduction

### Background

E-Learning and distance education are a growing part of postgraduate and continuous medical education. The cost effectiveness and logistical benefits have previously been shown [1] and, whereas 10 years ago e-learning was promising, it is now part of mainstream medical education [2]. However, the overall effectiveness and added value of e-learning over conventional education such as face-to-face learning is debatable, and results in the literature are diverse [3]. One of the problems in evaluating e-learning is the lack of a proper evaluation tool [4].

The effectiveness of e-learning can be separated into two parts: effect of the learning aim and instructional design of the e-learning module. By learning aim, we mean the ability of an e-learning module to achieve the learning goals, usually either new knowledge, skills, or attitude/behavior [5]. For example, the learning aim may be to tie a laparoscopic knot, which can be evaluated by an objective skill assessment tool. By instructional design, we mean the functionalities (affordances) and their design [6]. In the case of e-learning, these are the design of the digital medium and affordances used to achieve the learning aim—for example, the virtual reality program with interactivity, feedback, and gamification to practice the knot tying.

Despite the methodological limitations, e-learning is often evaluated by comparing test results before and after it is used. Usually, the learning aim is used as the primary outcome, and even when the design is also evaluated, this is generally done using instruments that are not aimed at postgraduate medical e-learning (PGMeL) [7]. When the design is not included in the e-learning evaluation study, however, the question arises about how to ascertain whether the e-learning modus is suited to the learning aim (eg, virtual reality is not suited for learning to tie a knot) or if there were essential flaws in the e-learning itself (eg, the virtual reality box was poorly designed). We believe that the evaluation of the learning aim should always go together with the evaluation of the design because they are interwoven in the final outcome. To properly evaluate the design, we need an instrument that has proper content validation and is aimed at the right target audience—in our case, PGMeL.

### Development of a Survey in Medical Education

**Figure 1:**
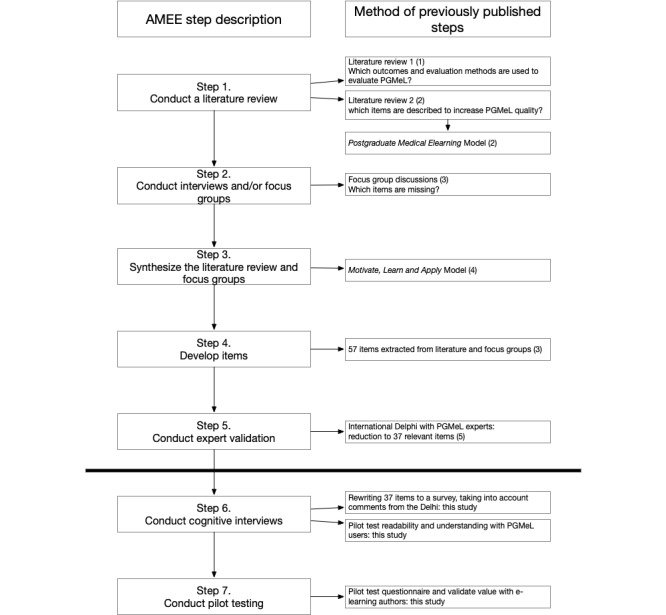
Association for Medical Education in Europe (AMEE) 7-step design process for developing surveys for medical education. PGMeL: postgraduate medical e-learning.

The development of an evaluation instrument is complex and involves many steps [8]. In 2014, the Association for Medical Education in Europe (AMEE) published a 7-step design process for developing surveys for medical education [9]. Steps 1 through 5 of the design were previously published in two reviews [7,10], focus group discussions [11], and an international Delphi study [4] (Figure 1). The aim of this study is to proceed with steps 6 and 7 and evaluate the results of the survey with the creators of a PGMeL. We want to know if the creators find the results helpful to improve the e-learning (usefulness), if they can understand the indicators that are used in the context of instructional design (understandability), and, finally, if the survey is offering them additional information over existing evaluation methods (added value). This leads to the following research question that this study will try to answer: Is a content-validated evaluation survey for PGMeL useful, understandable, and of added value for the creators of PGMeL?

## Methods

### Study Design

**Figure 2:**
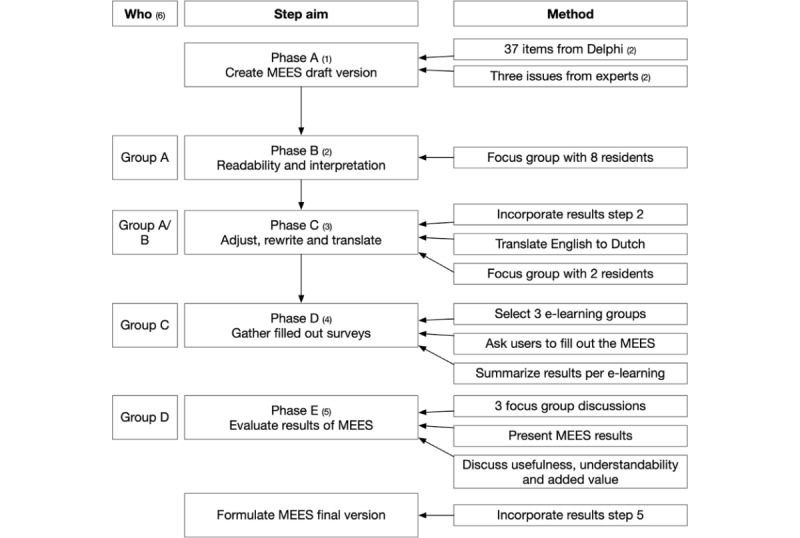
Five phases to address steps 6 and 7 of the Association for Medical Education in Europe design process and evaluate the usefulness, understandability, and added value of the Medical e-Learning Evaluation Survey (MEES).

To conclude the content validation, this study collected evidence of response process validity to assess how participants interpreted the items (AMEE step 6) and conducted pilot testing (AMEE step 7). To prevent confusion between the AMEE steps and the methodology of this study, we call the steps of this study phases. To answer the research question, this study has five phases, A through E (Figure 2).

- Create a survey draft based on 37 items of the previous Delphi study [4], and address three concerns of experts in this Delphi study. First, the term e-learning can be confusing; second, the added value of another survey might be limited; and third, the indicators may be too general for the evaluated e-learning module [4]. We called the survey the postgraduate Medical E-Learning Evaluation Survey (MEES).
- Determine readability and understandability with experienced postgraduate students by use of a focus group discussion.
- Adjust the survey draft according to the feedback in phase B. The English survey was also needed in Dutch (for phase D); therefore, the rewritten English survey was translated and again pilot tested for readability. Because less discussion was expected, only two (native Dutch) residents (not part of the first group of residents) were asked to read the Dutch survey and provide feedback.
- Use the survey by evaluating three PGMeL modules. Contacts of several European PGMeL groups were emailed and asked to participate, and the first three agreed. They were sent the participant information and the MEES. After agreeing to use the survey, they were asked to add it to the standard evaluation survey they might already have. Users participated voluntarily. Anonymized results of the surveys were sent to RDL.
- Perform focus group discussions with the creators of the e-learning modules about the survey results. The results were presented per domain. For each domain, the minimum, maximum, and average score; all items; and free text comments were discussed (Multimedia Appendix 1). Finally, a strength-weakness analysis with scores, summary of recognized items, and summary of free texts per domain were carried out. The discussion guide and short demographics questionnaire are in Multimedia Appendix 2.

We chose focus group discussions as the main methodology because they are an appropriate method to investigate attitudes and beliefs and generate new ideas [12].

### Study Participants

There were three groups of participants in this study. Group A members were experienced end users of PGMeL who participated in phase 1. These were OB/GYN (obstetrics and gynecology) residents in their fourth and fifth year at the Amsterdam University Medical Center. Group B members (also experienced residents) were asked to participate with the translation from English to Dutch. Residents were invited by RDL to participate by email and could decline without any consequences or repercussions.

The second group (group C) comprised users who undertook an e-learning module and were asked to complete the survey in phase C. This group was asked to evaluate the e-learning module they had just taken as part of the usual evaluation process. The e-module had to be (1) an approach aiming to teach and learn the adoption of new knowledge, skills, or attitude/behavior representing all or part of an educational model and (2) based on the use of electronic media and devices as tools to improve training access, communication, and interaction. The users had to be postgraduates in medicine and able to read and write in English or Dutch.

The third group (group D) were creators or authors of the e-learning modules described above. For phase D, we asked representatives tasked with the usual evaluation and improvement responsibilities of each evaluated module. The Dutch Association of Medical Education Research gave ethical consent (ID 2018.5.1).

### E-Learning Groups

This study evaluated three PGMeL modules. The aim was to gather survey outcomes to determine the usefulness, understandability, and added value of the modules with the creators. The aim was not to evaluate the modules themselves.

Module 1 was aimed at new doctors in a big teaching hospital in Amsterdam, the Netherlands. The e-learning aim was to train them to use the local electronic patient records. It was mandatory for all new doctors to complete the module, after which they were asked to complete the evaluation. The author group did not add extra items to the survey. A total of 160 participants were asked to fill out the survey from June to October 2018.

Module 2 was aimed at surgical residents and offered globally. The platform offered different surgical modules for a variety of specialties focusing on anatomy, surgical steps, and pitfalls during surgery. The author group added 19 extra items. After finishing at least three modules, users were asked to voluntarily complete the survey. From August to November 2018, 395 participants were asked to evaluate the e-learning modules.

Module 3 was aimed at OB/GYN residents practicing minimally invasive surgery, mainly in Europe. The e-learning module is part of a certification with face-to-face and hands-on training. The author group added 8 extra items. From August until the end of October 2018, about 2400 participants were asked to complete the survey. Of these, most were older users, some with email addresses that no longer worked. An estimated 1600 participants had recently used the e-learning module and were reached by email.

### Data Collection and Procedure

Data were collected in phases B and C by focus group discussion with experienced users in the comfort of their university environment. Data from phase D were collected by providing the MEES as an online survey with a short introductory text (Multimedia Appendix 3). Data for phase E were collected by audio recordings after written consent was given. These focus group discussions were facilitated by RDL at the main offices of the e-learning groups.

### Data Analysis

No analysis was undertaken of the first four phases as the data were used during the phases themselves. The focus group evaluations in phase E were analyzed. All interviews were transcribed verbatim and a thematic analysis was performed as per Braun et al [13]. The transcribing of the interviews was completed by RDL to enable the author to familiarize himself with the data. We used ATLAS.ti version 8.0 (ATLAS.ti Scientific Software Development GmbH) for the initial coding. Thematic analysis has been shown to usefully summarize key features and generate unanticipated insights [12,13]. To perform the analysis, we ordered the codes by the predefined themes from our research question: usefulness, understandability, and added value. We then reviewed the content of the themes and, if needed, redefined them as per Braun et al [13].

## Results

The results are described per phase in Figure 2. The initial draft and a change log of the MEES can be found online at www.MotivateLearnApply.com. The final version of the MEES is attached as Multimedia Appendix 3 and a creators’ manual is attached as Multimedia Appendix 4.

### Phase A: Postgraduate Medical E-Learning Evaluation Survey

To address expert feedback, we added an explanation to the survey and the option for e-learning creators to add additional items. The term e-learning was defined in a previous Delphi study and has been used for all studies so far: “an approach to teaching and learning, representing all or part of the educational model applied, that is based on the use of electronic media and devices as tools for improving access to training, communication, and interaction and that facilitates the adoption of new knowledge, skills, and/or behavior/attitude” [14]. This means that all forms of electronic learning based on an educational model are e-learning.

To address the generalizability of the indicators, they are used as examples. There are, for example, many ways to be motivated. The previous phases provided nine ways to achieve this, but the creators of the e-learning module might have used other strategies as well. Before the survey started, the creators of the modules were asked to add those indicators to each domain as examples. The domains were thus questioned using the general indicators from the literature but also items that might be unique to that one module.

**Figure 3:**
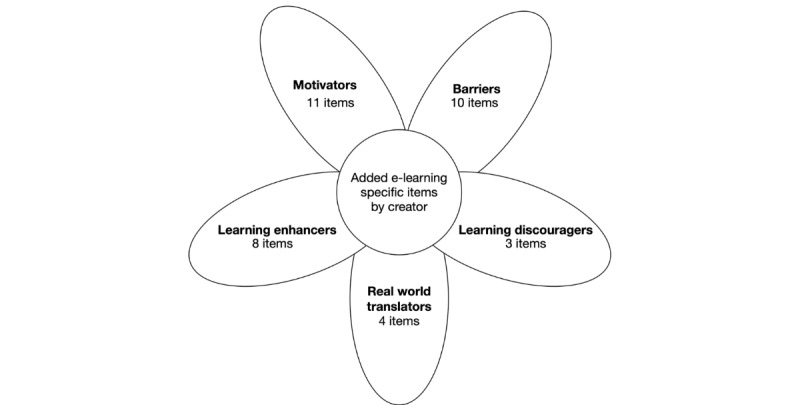
The domains and structure of the Medical e-Learning Evaluation Survey.

The MEES contains questions in five domains, each of which starts with a Likert scale of 1 to 10. These are followed by indicators from the previous phases and those added by the e-learning creators depending on the aims of the specific module. Finally, there is an open question about the domain. The domains are motivators, barriers, learning enhancers, learning discouragers, and real-world translators. The MEES contains 10 questions, 36 examples, and five open questions. Figure 3 shows the relationship between the domains, and Table 1 lists all 36 examples with a short explanation of the purpose.

**Table 1:** The 36 items from the final Medical E-Learning Evaluation Survey with a short explanation of their meaning from the creators’ points of view.

Domain and original item		Short explanation
**Motivators**		
	I felt this module was important.	Creating a feeling of importance is very important for the user. The challenge is to convey to your users that the learning aims are important for their work and personal development.
	I felt it was my responsibility to undertake this module.	Along with importance, your user needs to feel responsible for the learning aim as well. This can be done by emphasis on the importance, but also by, for example, rewarding or giving responsibility for an outcome.
	I had enough time to complete the module.	Proving time to do the module seems contradictory to “anytime, anywhere” learning, but it does give the learning the feeling of priority from a management level.
	I had a good understanding of the general purpose of the module.	The general purpose is the learning aim: knowledge, skills, or attitude/behavior. It should be very clear to the user what they gain from finishing the module.
	The e-learning objectives (for each educational section) were clear to me.	When a module is separated into different sections/chapters, make sure you communicate what the learning objectives are for each section.
	There was a clear overview of all content.	Providing an overview of all lessons, objectives, and options gives the user the possibility to manage expectations and, if possible, create their own learning process.
	I knew how to navigate to the content.	Navigation is an important part of the user interface and should be very clear for the user so they can find content easily and go back and forward through the content.
	I felt comfortable with the quality and truthfulness of the content.	Trust is important when learning. If the user has doubts about the truthfulness or quality, it will limit the working memory used for learning. Trust can be gained by the transparency of the creators, referring to recent literature, etc.
	I was able to undertake this module without being forced.	Forcing a user to undertake a module is the opposite of motivating them. If force or even blackmail is needed, the user will feel resentment, which kills motivation.
	I felt taken seriously as an adult learner.	Taking the learning seriously means avoiding childish illustrations or examples and aiming at the level of experience means that you take into account what the user already knows to prevent repetition of basic knowledge.
	The module was aimed at my level of experience.	Making your module too easy will decrease motivation, and making it too complicated will make users learn less. This is why knowing the background knowledge of the target audience is of great importance.
**Barriers**		
	I was not able to create my own learning path to my own needs.	This questions the difference between synchronized and asynchronized learning paths. Creating your own learning path means the option to test and skip already known sections or to go from A to C and then to B.
	The module was not easily accessible at my location or with my device.	Accessing the module should ideally be possible from every device and location, so consider, for example, internet speeds in foreign countries. If access is not possible, consider helping your users get the right device.
	The navigation did not make sense to me.	Good navigation is helpful but poor navigation will not only limit a module but make it impossible to finish. Make sure your users can follow all steps without using their cognitive load for navigation.
	The layout of the module was too complicated.	Navigation and layout are both important aspects of the user interface. The less cognitive energy is used for the learning environment, the more can be used for the learning itself.
	There was no instrument to help me navigate the module (eg, a sitemap).	Even if the navigation is of a high standard, it is still very helpful to have an instrument that gives an overview of all content and helps direct users where they want to be.
	I had concerns about the security and safety of the module regarding my personal information.	Worries about security and privacy are relevant in many countries and may even have a legal aspect.
	The module was slow and took too long to load.	Fast and logical use of the module is also an important aspect of the user experience. Waiting on affordances or loading frustrates and distracts and should be minimized.
	I did not know which devices the module was compatible with, and I might have used the wrong one.	If your module has specific needs (eg, a specific operating system such as iOS) you need to clearly state that at the beginning. Try to prevent users from experiencing your module in a wholly different way than planned because they use the wrong device.
	The module was too long.	The duration should have been specified. Duration of videos, sections, and the module overall are taken together as one item. If there are, for example, longer videos, their duration can be added as a separate item.
	The module did not divide the content into proper sections.	Learning and memory theories suggest that learning has a limited time span. Sectioning or chunking is a very effective way to help users through a bigger module.
**Learning enhancers**		
	I could personalize the module (eg, by saving and continuing, filling out questionnaires, and getting my personal score).	Personalizing a learning experience allows the user to know how they are doing and follow a preferred method and path. The more personal and specific such things as feedback are, the more the user will gain. This is a very important motivator as well.
	I could create my own learning path and was not forced to follow the directed path (eg, by skipping parts or returning to previous sections if needed).	This questioned the difference between synchronized and asynchronized learning paths. Creating your own learning path means the option to test and skip already known sections or to go from A to C and then to B.
	I had an idea of the progress I had made and what was left to do (eg, by a progress bar).	When learning, it’s important to manage expectations. Knowing what is already done and what is left to do is an important affordance of, for example, a book, and should preferably be available in a module as well.
	I had access to technical support if needed.	To minimize the effort spent on technical aspects rather than learning, providing support as fast as possible will prevent users from stopping learning.
	The module provided summaries where needed.	Learning theory suggests that summaries support learning by offering repetition of content in a new format and allowing chunking of the bigger picture.
	The module provided feedback on my answers.	Learning theory also suggests that learning is more effective when based on previous experience and knowledge, and providing feedback helps the user to make connections between new knowledge and their mistaken or correct assumptions.
	There were exercises and/or assignments in the module.	Learning theory suggests that actively using new knowledge will help it to go from working memory to long-term memory. Therefore, exercises or assignments help the transfer of the learning aim to long term memory.
	I could interact with the content of the module (eg, questions, exercises, or other interactivities).	Interaction is another example of actively using the content, helping users learn more efficiently.
**Learning discouragers**		
	I got stressed or frustrated by the module for whatever reason.	Stress can be caused by many things but will always distract from learning. Stress can come from failing hardware, deadlines, the consequences of failing, etc.
	The content was not able to adapt to my device when needed (eg, the module should work on a mobile device, but the icons were much too small for that).	Nonadaptable content can cause frustration and degrade the user experience, again moving energy away from learning and toward technical aspects.
	The e-learning design and visuals were too distracting for me.	Multimedia learning provides a theory and guidelines for how to use the combination of visuals and auditory stimuli effectively. Distraction should always be prevented.
**Real-world translators**		
	The e-learning content and examples are translatable to my daily real-world work.	Adult learning theory suggests that adults prefer learning in a professional environment, if they can use the lessons learned in daily practice. Providing content and examples that are relatable will help.
	The module seems up to date and properly maintained.	When a user thinks they are learning old material, it might not seem applicable to their daily work anymore. This will kill motivation and minimize the effort the user is willing to put in.
	The module provided sources for the information that were also accessible after finishing it.	Health care professionals in particular might want to undertake further reading in a relevant topic or refresh their memory after finishing the module. Providing this and letting the user know this is possible will increase motivation.
	Besides this questionnaire, the module was evaluated on topics like user experience, effectiveness, usability, and/or costs.	The literature suggests that evaluation is an important step. This question is an oxymoron because by asking it, you are already evaluating. Therefore, the question is: Are OTHER evaluation instruments ADDED to this evaluation—for example, focus group discussions?

### Phase B: Readability and Item Interpretation

Eight residents were asked to participate and all agreed, but one was unable to come to the discussion on time. The discussion lasted 65 minutes and took place in May 2017 at the Amsterdam University Medical Center, the Netherlands. Four items were interpreted differently than intended, three items were not readable, and two items were in two domains. Details can be found in the change log online. Overall, domains and questions were well understood.

### Phase C: Adjust, Rewrite, and Translate

The items from phase A were rewritten. After they had been adjusted, they were understood correctly. After finishing the English MEES, RDL translated the survey into Dutch. Two native Dutch residents from the Amsterdam University Medical Center read the survey in May 2017. All items were clear and were understood correctly. No other changes were made. The MEES version used for the evaluation contained three positive domains (motivation, learning enhancers, and real-world translation) and two negative domains (barriers and learning discouragers), with 36 items in those domains, 5 Likert scale questions of 1 to 10, and 5 open questions asking the participants their own comments in each domain. Figure 3 provides an overview of the domains and number of questions per domain in the MEES survey.

### Phase D: Gathering Completed Surveys

Details of the three evaluated modules are summarized in Table 2, and the scores per domain are in Table 3. All evaluations took place between July and the end of November 2018, after which we concluded the evaluation.

The higher the score in the positive domains (motivators, learning enhancers, and real-world translators), the better. In the negative domains barriers and learning discouragers, a lower score is better. Note the discrimination between the positive and negative domains.

In total, there were 77 free text comments (see Multimedia Appendix 1 for all comments). The main positive comments concerned the availability and added value of the module to local education.

Unfortunately, surgical skills at my university are not well taught due to the large number of residents. There is also lack of standardization in teaching. I was happy to find a fun way to learn the best, standard way to perform common gynecological procedures and no longer rely on sketchy YouTube videos.

On the negative side, users complained mostly about technical barriers such as long loading times, log-in problems, software crashes, and nonfunctioning affordances such as search functions.

It sometimes took too long to load despite good internet connection, and I have often been forced to abandon a procedure due to this.

Video streaming is a serious limitation. There is a need for video downloads.

Another frequent complaint was about the language barriers such as poor English or videos with hard-to-understand speakers.

Difficulte de langue. Je ferais effort d’aprendre.

**Table 2:** Summary details of the three evaluated modules. For more details, see Multimedia Appendix 1.

Module	Target audience	Location	Additional items	Survey status	
				Invited, n	Completed, n (%)
1	Medical staff	Netherlands	0	160	16 (10.0)
2	Residents	Worldwide	19	395	36 (9.1)
3	OB/GYN^a^ residents	Worldwide	8	1600	106 (6.6)

^a^OB/GYN: obstetrics and gynecology.

**Table 3:** Domain scores (range 1-10) per domain of the three evaluated modules.

Module	Motivator, median (IQR^a^)	Barrier, median (IQR)	Learning enhancer, median (IQR)	Learning discourager, median (IQR)	Real-world translator, median (IQR)
1	7.5 (2.0)	3.0 (3.8)	7.0 (2.0)	4.0 (2.0)	8.0 (3.0)
2	9.0 (2.3)	3.0 (3.0)	9.0 (3.0)	3.0 (3.0)	8.5 (3.0)
3	10.0 (2.0)	2.0 (5.0)	9.0 (2.0)	1.0 (4.0)	9.0 (2.0)

^a^IQR: interquartile range.

### Phase E: Focus Group Discussions With the E-Learning Creators

Three focus group discussions took place, one with each e-learning creator group at their main office in November and December 2018. The average age of the 10 participants was 51 years with participants having 0 to 5 years’ experience in creating e-learning, and there were content, didactic, and technical experts at the interviews. While 80% (8/10) had experience with previous evaluations, only 10% (1/10) had used any formal evaluation methods. The three subjects of discussion are now described.

Usefulness: all participants described the results as very useful.

Grouping the results into positive (domains) and negative (domains) resulted in a clear overview of what we need to keep and what to improve.

1B

All groups said that the option to add items specific to a module increases the usefulness. It provides feedback on the additional items that are considered important for the creator group. The first group regretted not adding any items themselves and, seeing the results now, said they would have added them.

Understandability: going over the items one by one, some were not clear to the creators. Even though they understood the question, they did not know how to interpret it from a creator’s point of view. These items (10, 12, and 36) are in Multimedia Appendix 4. The general advice was to have an explanation manual for the creators of each item that could be consulted in the event of misunderstanding.

It would be nice to have a short explanation per item.

2C

There were also worries about the form in which the results were presented. All the completed surveys were presented by RDL and summarized by him as well.

How much time did it take you to formulate the results like this, and can this be done by us as well?

3A

Added value: although the participants had experience evaluating e-learning modules, only one had used a formal evaluation method (although it is unknown which one). There were three subjects that added value to the MEES, the first of which was the domains. Using the domains gave the creators a structure that they did not have with other, informal, evaluation methods. Second, the items provided concrete examples of do’s and don’ts, which inspired the changes needed in an update. Third, using a formal method gave the creators a feeling of importance and allowed them to formalize the needs for improvement. The creators believed that using a formal method would allow them to more easily convince management and increase the commercial benefits of the module.

This really helps to improve the commercial value of our e-learning.

2A

The five phases of this study provided a first draft of the MEES, an adjusted version (Multimedia Appendix 3), evaluation data from three PGMeL modules (Multimedia Appendix 1), and the thematic analysis of three focus group discussions with the creators of these modules.

## Discussion

### Principal Findings

To our knowledge, the MEES is the first survey designed to evaluate PGMeL. Content validation has been completed, and this study completes all seven steps described by the AMEE. We set out to investigate the usefulness, understandability, and added value of this survey with the creators of three PGMeL modules. This study shows that the MEES is very useful, that understandability is clear with the help of a creators’ manual (Multimedia Appendix 4), and that the MEES is of added value in connection with the structured domains and validated nature of the survey. Although the MEES has reached a second stage, two questions remain.

First, how do we know the items have been correctly understood by all the participants? The pilot evaluation was carried out with Dutch participants who seemed to understand the questions in a group session. That might be generalizable to Dutch residents. However, the second and third evaluated e-learning modules involved European and worldwide users who might not have interpreted the questions correctly. To then interpret an evaluation, it is important to at least consider the content integration and evaluate the equity pedagogy [15]. Content integration means using content that will illustrate the same examples in a variety of cultures. Equity pedagogy exists when the curriculum is modified to accommodate and facilitate the academic achievements of a diverse group. Although debatable, using the cultural dimensions of Hofstede [16] can also help to place extreme high or extreme low scores in a cultural light. For example, in a culture with high power distance (in other words, where power and inequality are fundamental facets of the society), users might be tempted to express gratitude by providing extremely positive feedback.

Second, how many completed surveys are needed for a proper evaluation? Ideally, you would ask participants to keep completing the survey until a theoretical data saturation is met. This would require data analysis, after, for example, every tenth survey. But as seen above, the average reply was around 8%; thus, 92% of users are missed when reply is made voluntary. Besides the practical problem of determining data saturation, replies stopped whether you need more or not. This can be increased by, for example, asking respondents to fill out a survey before supplying a needed certificate. This might raise an ethical dilemma in a research setting like this. For this study, the response rate was less relevant. We were not aiming to evaluate the e-learning modules, we were aiming to evaluate the outcome with the creators of the modules. When using this survey in practice, the authors should consider making it mandatory to get a higher response rate. Other ways to assemble a representative group include purposive sampling [17], but the question remains as to how many is enough. The conclusion is that the more respondents, the better the data, but the reality is that researchers can only work with the data made available to them.

On the other hand, although missing 92% of users might seem to indicate a failed evaluation, the question of who is most likely to complete the survey is a valid one. Hu et al [18] show that users who provide feedback are most likely to be either very satisfied or very disappointed and thus may be exactly the respondents required; that is, it may be that the middle group yields less information of use to the evaluation.

### Strengths and Limitations

Following all steps of the AMEE guide and peer publishing them might be the biggest strength of the MEES. Questionnaire validation is a complex and diverse field of expertise, and this study takes an important next step. An international Delphi study in 2010 (Consensus-Based Standards for the Selection of Health Status Measurement Instruments) helped to structure the diverse terminology within this field by providing this definition: “validity is the degree to which an instrument truly measures the constructs it proposes to measure” [8]. Many types of validity can be evaluated, and validation is a continuous process. A short list, partly based on Tsang et al [19], of forms of validation is given in Table 4 with an explanation of their applicability to the MEES. It can be seen that face and content validity are completed and that construct, criterion, and predictive validity leave room for future research. Because structural and concurrent validity are not applicable, factor analysis was deliberately not performed.

**Table 4:** Forms of validation of a survey in relation to the Medical E-Learning Evaluation Survey.

Type of validation	Explanation	Relationship to MEES^a^
Face	Whether the instrument is understandable and relevant	Checked in this study by asking the PGMeL^b^ creators about understandability and added value
Content	Whether the instrument measures the most important aspects of a concept that it is designed to evaluate	Checked in three previous studies by means of a review [10], focus groups [11], and an international Delphi study [4]
Construct	The degree to which the instrument’s scores relate to other measures in a manner that is consistent with an a priori hypothesis concerning the concepts being measured	Awaiting future validation: the construct of the MEES is predicting efficiency and effectiveness by evaluating the experience of affordances to determine the quality of the instructional design
Criterion	How well one measure predicts the outcome of another	Awaiting future validation: MEES might predict the satisfaction or learning aim transference of the PGMeL
Predictive	The instrument’s ability to predict future test results	Awaiting future validation: as regards the MEES, this is in line with the criterion validity and can be checked in the future
Structural	Whether all items in a scale or subscale measure the same concept of the dimensionality of the instrument	Not applicable; this can be done by factor analysis but assumes that a scale or subscale is highly correlated, which might not be the case in the MEES
Known-group	The ability to be sensitive to differences between groups of users that may be expected to score differently in the predicted direction	Not applicable; this can be done by comparing PGMeL designed for certain cultural groups, but the assumptions are too complex to use this in practice
Concurrent	The association of the instrument with accepted standards	Not applicable; there is no gold standard of evaluating PGMeL

^a^MEES: Medical E-Learning Evaluation Survey.

^b^PGMeL: postgraduate medical e-learning.

Another strength lies in the involvement of end users. By including experienced residents in the focus group discussions, Delphi surveys, and pilot evaluation, we believe the MEES is truly a user-centered method of evaluation as it evaluates not only theoretical items but also subjects that matter to the user. The numbers from the Likert scale questions do not offer enough insight, but the free texts fields do add knowledge about the users’ needs and wishes. Most of the comments concerned e-learning affordances and technical execution (eg, interactive videos that load too slow). This emphasis shows the importance of evaluating not only the content of the module but the instructional design as a whole.

The biggest limitation of the MEES is also addressed above. It is not possible to know what feedback has been missed from those users who did not fill out the form. This might be reduced by making an evaluation mandatory, but it is impossible to predict how motivated users will be to provide useful feedback. We therefore believe that the MEES should always be accompanied by in-depth focus group evaluations with the users. Not only will these provide missed feedback, but they will also allow researchers to find out why some items are recognized or missed. Proper evaluation should never contain only an online survey.

### Future Research

Validation is a never-ending story, and it is necessary to continue collecting validity evidence [9]. We believe three steps should be taken. First, as part of the construct and criterion validation, it would be interesting to see if the MEES can be used to measure improvement by taking a PGMeL, evaluating it with the MEES and a focus group discussion, adjusting it accordingly, and reevaluating to see if the second evaluation is better. This would provide insight into the actual benefit for future learning. The second step would be to evaluate readability and understandability in different languages. To this end, the survey should be translated into other languages and those new translations pilot-tested for readability and understandability.

The third step is to evaluate the understandability and reliability of the survey within different subcultures. Evaluating how different cultural groups interpret digital evaluation and the questions can provide insight into the way the creators should use the results of the survey.

### Conclusion

This study provides the first instructional design evaluation survey for postgraduate medical e-learning. Content validation has been completed, and this study completes all seven steps described by the AMEE for the development of an evaluation instrument for medical education. The survey was experienced as useful, understandable, and of added value. Future research can continue the validation process and follow up in the daily practice of evaluating e-learning.

## Appendix

Multimedia Appendix 1 E-Learning survey outcomes.

Multimedia Appendix 2 Focus group discussion guide.

Multimedia Appendix 3 Final version of the Medical E-Learning Evaluation Survey.

Multimedia Appendix 4 Creators’ manual.

## Abbreviations

PGMeL: postgraduate medical e-learning
AMEE: Association for Medical Education in Europe
IQR: interquartile range
MEES: Medical E-Learning Evaluation Survey
OB/GYN: obstetrics and gynecology
