# Supplementary material for: Development of an Instructional Design Evaluation Survey for Postgraduate Medical E-Learning: Content Validation Study
Source: J Med Internet Res. 2019 Aug 9;21(8):e13921. doi: 10.2196/13921 (PMC6713039; doi:10.2196/13921)
Supplement: Multimedia Appendix 1 [file jmir_v21i8e13921_app1.docx]

# Appendix 1 – e-learning survey outcomes

E-learning 1: twenty-three additional comments were given in the open questions. Eight items of the positive domains were recognized by at least half the participants and none of the items in the negative domains.

E-learning 2: Most of the residents were from general surgery (n=19) and from the Netherlands (n=8) and Indonesia (n=4). Ten items from the positive domains and one from the negative were recognized. Twenty-four remarks were left in the free text fields.

E-learning 3: Ten items in the positive domains were recognized and none in the negative domains. Thirty free text comments were left.

Table 1 shows all scores per e-learning and per domain. Table 2 provides all the original free text comments and can be found online at <https://www.MotivateLearnApply.com>

*Table 1: scores per e-learning, per domain*
