# Supplementary material for: Development of an Instructional Design Evaluation Survey for Postgraduate Medical E-Learning: Content Validation Study
Source: J Med Internet Res. 2019 Aug 9;21(8):e13921. doi: 10.2196/13921 (PMC6713039; doi:10.2196/13921)
Supplement: Multimedia Appendix 2 [file jmir_v21i8e13921_app2.docx]

# Appendix 2 – focus group discussion guide

## Introduction

We met today to look at the results of the previously completed questionnaire. You already had these results before and the goal today is to review these results once again and then evaluate together which aspects were of value to you. It is not about the evaluation outcomes themselves. So, we are not going to look at the strengths and improvements of your e-learning. We will look at the strengths and weaknesses of the evaluation survey itself, for you as a creator. We will do this by category: motivation, learning and applicability.

## A. Interview background

Would you please fill out the demographic questionnaire below?

## B. Domain motivation

After this brief overview of the results, did you find these results useful?

Did you understand all the items questioned?

Which aspects come from the EMSE are not of added value?

Which aspects would you miss in the evaluation of this domain?

## C. Learn domain

After this brief overview of the results, did you find these results useful?

Did you understand all the items questioned?

Which aspects come from the EMSE are not of added value?

Which aspects would you miss in the evaluation of this domain?

## D. Domain applicability

After this brief overview of the results, did you find these results useful?

Did you understand all the items questioned?

Which aspects come from the EMSE are not of added value?

Which aspects would you miss in the evaluation of this domain?

## E. General comments

Many thanks for answering these questions. Are there any general things that you think can improve MEES in any way?

## Demographic questions

1. What is your age? ____________________
2. What is your responsibility in the e-learning creation process?
   1. Content expert
   2. Didactic / education expert
   3. Technical expert
   4. Other ______________________
3. How many years of experience do you have with e-learning?
   1. 0-5 years
   2. 5-10 years
   3. > 10 years
4. Do you have experience in the formal evaluation of e-learning?
   1. Yes
   2. No
5. If yes, how many e-learnings did you evaluate approximately?
   1. 0-5
   2. 5-10
   3. >10
6. If yes, did you use any validated evaluation instruments?
   1. Yes
   2. No
7. If yes, which one? _________________________
