# Supplementary material for: Development of an Instructional Design Evaluation Survey for Postgraduate Medical E-Learning: Content Validation Study
Source: J Med Internet Res. 2019 Aug 9;21(8):e13921. doi: 10.2196/13921 (PMC6713039; doi:10.2196/13921)
Supplement: Multimedia Appendix 3 [file jmir_v21i8e13921_app3.docx]

# Appendix 3 – final MEES version

Dear e-learning creator.,

This survey is aiming to evaluate the quality of the instructional design of postgraduate medical e-learning by evaluating the experience of the affordances of your e-learning, aiming to predict the efficiency and effectiveness.
There are 10 questions, 36 examples and 5 open questions already in the survey. You are asked to add examples, depending on your learning aims and unique affordances. Please find a detailed description of the way to use this survey and how to interpret all examples in the attached “Creator manual”. An example text for your users and a short summary example text for the documentation of the evaluation can also be found in this manual.

The Evaluation Survey for postgraduate Medical E-learning (MEES) version 1.0-2019

[please add your own introduction text here, or find an example in the creators’ manual]

- 1. On a scale of 1-10, how motivated were you to start the e-learning?

Unmotivated Motivated

- 1. Please select which of the following items motivated you (multiple options).
- I felt this e-learning was important
- I felt it was my responsibility to do this e-learning
- I had enough time to do the e-learning
- I had a good understanding of the general purpose of the e-learning
- The e-learning objectives (for each educational section) were clear to me
- There was a clear overview of all content
- I knew how to navigate to the content
- I felt comfortable with the quality / truthfulness of the content
- I was able to do this e-learning unforced
- I felt taken seriously as an adult learning
- The e-learning was aimed at my level of experience
- ADDED EXAMPLE(S)
- Please write down any other item(s) that motivated you:
  1. On a scale of 1-10, did you experience any barriers to starting the e-learning?

Stimulants Barriers

1.4 Please select which of the following items you experienced as a barrier to starting the e-learning.

- I was not able to create my own learning path to my own needs
- The e-learning was not easily accessible at my location or with my device
- The navigation did not make sense to me
- The layout of the e-learning was too complicated
- There was no instrument to help me navigate the e-learning (for example a sitemap)
- I had worries about the security and safety of the e-learning, regarding my personal information
- The e-learning was slow and took too long to load
- I did not know which devices the e-learning was compatible with and I might have used the wrong one
- The e-learning was too long
- The e-learning did not divide the content into proper sections
- ADDED EXAMPLE(S)
- Other item(s) that I experienced as a barrier:

2.1 On a scale of 1-10, how educative was the e-learning for you?

Uninformative Educative

2.2 Please select which of the following 8 items helped you to learn and remember or add your own.

- I could personalize the e-learning (for example by saving and continuing, filling out questionnaires and getting my personal score, etc)
- I could create my own learning path, and was not forced to follow the directed path (for example by skipping parts or returning to previous sections if needed)
- I had an idea of the progress I had made and what was left to do (for example by a progress bar)
- If needed, I had access to technical support
- The e-learning provided summaries where needed
- The e-learning provided feedback on my answers
- There were exercises and/or assignments in the e-learning
- I could interact with the content of the e-learning (for example questions, exercises or other interactivities)
- ADDED EXAMPLE(S)
- Other item(s) that helped me learn and remember:

2.3 On a scale of 1-10, did you experience limitations in the e-learning that prevented you learning?

Advantages Limitations

2.4 Please select which of the following 3 limitations you experienced or add your own.

- I got stressed or frustrated by the e-learning for any reason
- The content was not able to adapt to my device when needed (for example, the e-learning should work on a mobile device, but the icons were way too small for that)
- The e-learning design and visuals were too distracting for me
- ADDED EXAMPLE(S)
- Other limitation(s) in my learning from this e-learning:

3.1 On a scale of 1-10, how do you think you can apply the newly learned knowledge, skills or attitude to your daily work?

Irrelevant Applicable

3.2 Please select which of the following 4 items helped you to apply the e-learning, or add your own.

- The e-learning content and examples are translatable to my daily real-world work
- The e-learning seems up-to-date and maintained
- The e-learning provided sources for the information which were also accessible after finishing it
- Besides this questionnaire, the e-learning was evaluated on topics like user experience, effectiveness, usability and/or costs
- ADDED EXAMPLE(S)
- Another way this e-learning helped me apply newly learned knowledge, skills or attitude to my daily work:
