# Supplementary material for: Development of an Instructional Design Evaluation Survey for Postgraduate Medical E-Learning: Content Validation Study
Source: J Med Internet Res. 2019 Aug 9;21(8):e13921. doi: 10.2196/13921 (PMC6713039; doi:10.2196/13921)
Supplement: Multimedia Appendix 4 [file jmir_v21i8e13921_app4.docx]

# Appendix 4 - Creators’ Manual

This is a Postgraduate Medical e-learning (PGMeL) Creators’ Manual which should be used with the Evaluation Survey for Postgraduate Medical E-learning (MEES).

This survey is publicly available and was published in 2019 in XXX, with the title “The development and validation of an Evaluation Survey for Postgraduate Medical E-learning”. It can be freely used, and all questions may be addressed to the corresponding creator of the article.

The aim of the survey is to evaluate the quality of the instructional design of PGMeL by evaluating the experience of the affordances of an e-learning with the aim of predicting its efficiency and effectiveness.

## Table of content

1. Using the survey
2. Definitions
3. All items explained
4. Using the results
5. Publishing the results

## Using the survey

Before digitalizing the survey, you should first consider each domain for your own e-learning. Each domain is described below and will contain three question types: first, a Likert-scale of 0-10; then, a series of literature-based examples which your users may or may not recognize. Because each e-learning is unique, you are asked to think about the unique characteristics or functionalities of yours. Try to place them in the domains and add them to the list of examples. Each domain ends with an open question, asking the learners what they think can be added or removed from the list.

When you have done that, we advise you to digitalize the survey and add it to the end of your e-learning. It can be made mandatory, which might elicit many neutral and unmotivated responses, or voluntary, which will give you fewer and usually more polarized opinions. Our experience is that the latter provides more useful feedback.

## Definitions

To understand the explanations of the survey, we have provided you below first with a list of used definitions and then with definitions of the used domains.

Definitions:
The user the person using and learning from your e-learning

The creator the person, or more usually the team, that created the e-learning. (We did not use the word ‘author’ to prevent confusion with the author of a manuscript)

User interface all components of the e-learning that make interaction between human and hardware/software possible, for example a menu

User experience the relation between the e-learning and the user: which emotions and feelings – for example, pleasure or frustration – they experienced during the use of your e-learning

Affordances the functionalities and options that your e-learning provides to the user in order to make the experience as efficient and effective as possible, for example feedback on incorrect questions

Instructional design the way your e-learning is designed; which educational strategies are used; and which affordances are chosen and executed in order to make the e-learning

Cognitive load a part of the cognitive load theory, a learning theory that provides a model of how people learn. Many aspects of the evaluation are based on this theory and further reading is advised, for example *Cognitive load theory in health professional education: design principles and strategies,* van Merriënboer et al. *Medical Education* 2010: 44: 85–93

Multimedia learning a learning model from Mayer et al providing a list of guidelines to make use of the cognitive load theory by visual and auditory means. Further reading*: The Cambridge Handbook of Multimedia Learning*. R.E. Mayer. Cambridge Press: New York 2014.

## The domains:

Motivators motivators are aspects of the e-learning that will help the user start the e-learning and continue it to the end

Barriers the opposite of the motivators domain; things that will prevent the user from starting or finishing the e-learning. There is a scale with motivators on one hand and barriers on the other

Learning enhancers affordances that will, based on learning theories, increase the efficiency of the learning process of the user

Learning discouragers aspects that will distract or demotivate the user, or wrongly guide their learning

Real-world translators aspects of the e-learning that will help the user to apply the new knowledge, skills and/or behaviour in their daily work

## Table 1 - All items explained per domain

| **Domain** |  | **Original item** | **Short explanation** |
| --- | --- | --- | --- |
| Motivate | 1 | I felt this e-learning was important | Creating a feeling of importance is very important for the user. The challenge is to convey to your users that the learning aims are important for their work and personal development |
|  | 2 | I felt it was my responsibility to undertake this e-learning | Along with importance, your user needs to feel responsible for the learning aim as well. This can be done by emphasis on the importance, but also by, for example, rewarding or giving responsibility for an outcome |
|  | 3 | I had enough time to complete the e-learning | Proving time to do the e-learning seems contra to "anytime, anywhere" learning, but it does give the learning the feeling of priority from a management level |
|  | 4 | I had a good understanding of the general purpose of the e-learning | The general purpose is the learning aim: knowledge, skills or attitude/behaviour. It should be very clear to the user what they gain from finishing the e-learning |
|  | 5 | The e-learning objectives (for each educational section) were clear to me | When an e-learning is separated into different sections / chapters, make sure you communicate what the learning objectives are for each section |
|  | 6 | There was a clear overview of all content | Providing an overview of all lessons, objectives and options gives the user the possibility to manage expectations and if possible, create their own learning process |
|  | 7 | I knew how to navigate to the content | Navigation is an important part of the User Interface and should be very clear for the user so they can find content easily and go back and forward through the content |
|  | 8 | I felt comfortable with the quality / truthfulness of the content | Trust is important when learning. If the user has doubts about the truthfulness or quality, it will limit the working memory used for learning. Trust can be gained by the transparency of the creators, referring to recent literature, etc. |
|  | 9 | I was able to undertake this e-learning without being forced | Forcing a user to undertake an e-learning is the opposite of motivating them. If force or even blackmail is needed, the user will feel resentment, which kills motivation |
|  | 10 | I felt taken seriously as an adult learning | Taking the learning seriously means avoiding childish illustrations and/or examples, and aiming at the level of experience means that you take into account what the user already knows to prevent repetition of basic knowledge |
|  | 11 | The e-learning was aimed at my level of experience | Making your e-learning too easy will decrease motivation, while making it too complicated will make users learn less. This is why knowing the background knowledge of the target audience is of great importance |
| Barriers | 12 | I was not able to create my own learning path to my own needs | This questions the difference between synchronized and asynchronized learning paths. Creating your own learning path means the option to test and skip already known sections or to go from A to C and then to B |
|  | 13 | The e-learning was not easily accessible at my location or with my device | Accessing the e-learning should ideally be possible from every device and location so consider, for example, internet speeds in foreign countries. If access is not possible, consider helping your users get the right device |
|  | 14 | The navigation did not make sense to me | Good navigation is helpful but poor navigation will not only limit an e-learning, but make it impossible to finish. Make sure your users can follow all steps without using their cognitive load for navigation |
|  | 15 | The layout of the e-learning was too complicated | Navigation and layout are both important aspects of the User Interface. The less cognitive energy is used for the learning environment, the more can be used for the learning itself |
|  | 16 | There was no instrument to help me navigate the e-learning (for example a sitemap) | Even if the navigation is of a high standard, it is still very helpful to have some instrument that gives an overview of all content and helps direct users where they want to be |
|  | 17 | I had worries about the security and safety of the e-learning, regarding my personal information | Worries about quality are mentioned in item 8, but worries about security and privacy are also relevant in many countries, and may even have a legal aspect |
|  | 18 | The e-learning was slow and took too long to load | Fast and logical use of the e-learning is also an important aspect of the User Experience. Waiting on affordances or loading frustrates and distracts, and should be minimized |
|  | 19 | I did not know which devices the e-learning was compatible with and I might have used the wrong one | If your e-learning has specific needs, for example a specific operating system such as iOS, you need to clearly state that at the beginning. Try to prevent users from experiencing your e-learning in a wholly different way than planned because they use the wrong device |
|  | 20 | The e-learning was too long | The duration should have been specified. Duration of videos, sections and the e-learning overall are taken together as one item. If there are, for example, longer videos, their duration can be added as a separate item |
|  | 21 | The e-learning did not divide the content into proper sections | Learning and memory theories suggest that learning has a limited time span. Sectioning or chunking is a very effective way to help users through a bigger e-learning |
| Learning enhancers | 22 | I could personalize the e-learning (for example by saving and continuing, filling out questionnaires and getting my personal score, etc) | Personalizing a learning experience allows the user to know how they are doing and follow a preferred method and path. The more personal/specific such things as feedback are, the more the user will gain. This is a very important motivator as well |
|  | 23 | I could create my own learning path, and was not forced to follow the directed path (for example by skipping parts or returning to previous sections if needed) | This questioned the difference between synchronized and asynchronized learning paths. Creating your own learning path means the option to test and skip already known sections or to go from A to C and then to B |
|  | 24 | I had an idea of the progress I had made and what was left to do (for example by a progress bar) | When learning, it's important to manage expectations. Knowing what is already done and what is left to do is an important affordance of, for example, a book, and should preferably be available in an e-learning as well |
|  | 25 | If needed, I had access to technical support | To minimize the effort spent on technical aspects rather than learning, providing support as fast as possible will prevent users from stopping learning |
|  | 26 | The e-learning provided summaries where needed | Learning theory suggests that summaries support learning by offering repetition of content in a new format and allow chunking of the bigger picture |
|  | 27 | The e-learning provided feedback on my answers | Learning theory also suggests that learning is more effective when based on previous experience and knowledge, and providing feedback helps the user to make connections between new knowledge and their mistaken or correct assumptions |
|  | 28 | There were exercises and/or assignments in the e-learning | Learning theory suggests that actively using new knowledge will help it to go from working memory to long-term memory. Therefore, exercises or assignments help the transfer of the learning aim to long term memory |
|  | 29 | I could interact with the content of the e-learning (for example questions, exercises or other interactivities) | Interaction is another example of actively using the content, helping users learn more efficiently |
| Learning discouragers | 30 | I got stressed or frustrated by the e-learning, for whatever reason | Stress can be caused by many things, but will always distract from learning. Stress can come from failing hardware, deadlines, the consequences of failing, etc |
|  | 31 | The content was not able to adapt to my device when needed (for example, the e-learning should work on a mobile device but the icons were much too small for that) | Non-adaptable content can cause frustration and degrade the User Experience, again moving energy away from learning and towards technical aspects |
|  | 32 | The e-learning design and visuals were too distracting for me | Multimedia learning provides a theory and guidelines for how to use the combination of visuals and auditory stimuli effectively. Distraction should always be prevented |
| Real-world translators | 33 | The e-learning content and examples are translatable to my daily real-world work | Adult learning theory suggests that adults prefer learning in a professional environment, if they can use the lessons learned in daily practice. Providing content and examples that are relatable will help with that |
|  | 34 | The e-learning seems up-to-date and properly maintained | When a user thinks they are learning old material, it might not seem applicable to their daily work anymore. This will kill motivation and minimize the effort the user is willing to put in |
|  | 35 | The e-learning provided sources for the information which were also accessible after finishing it | Health care professionals in particular might want to undertake further reading in a relevant topic, or refresh their memory after finishing the e-learning. Providing this and letting the user know this is possible will increase motivation. |
|  | 36 | Besides this questionnaire, the e-learning was evaluated on topics like user experience, effectiveness, usability and/or costs | The literature suggests that evaluation is an important step. This question is an oxymoron because by asking it, you are already evaluating. Therefore, the question is: are OTHER evaluation instruments ADDED to this evaluation, for example focus group discussions? |

## Using the results

After gathering a sufficient sample of filled out surveys, there are many ways to use them. One way of doing so is by creating a strength and weakness analysis.

1. Gather the highest, lowest and mean score. Having outliers can be interpreted as a good thing, as it means you have the opinions of very satisfied but also dissatisfied users.
2. The strengths are in the motivation, learning enhancers and real-world translation domains. Items that are recognized by under 50% of users might need more attention. Use the free text comments as inspiration for further development.
3. The weaknesses are in the barriers and learning discouragers domains. When more then 50% of users do recognize an item, it might need to be removed. Use the free text to determine what you recognize, as well, and try preventing such items in the next version.

An example of such a strength and weakness analysis can be found online at [www.motivatelearnapply.com](http://www.motivatelearnapply.com)

## Publishing the results

We encourage creators to publish the results of the MEES. This will show the users that their effort is actually being put to good use, which will motivate future evaluations. It also shows that you are serious in wanting to improve and continue the e-learning, which will increase the trust the users have in it.

Publishing can mean publishing the mean scores, examples recognized by at least 50% and a short plan of improvement for future versions. Ideally, you should indicate a schedule for when this will take place, so users know when to return for a new version as well.

The use of the MEES can be justified by the following text:

“We used a validated questionnaire called the Evaluation Survey for Postgraduate Medical E-learning (MEES) to evaluate our e-learning. This survey is developed specifically for PGMeL and is based on five publications in peer reviewed medical journals. The version we used is published in XXX, with the title “*The development and validation of an Evaluation Survey for postgraduate Medical E-learning“* by de Leeuw et al in 2019.

The aim of the survey is to evaluate the quality of the instructional design of PGMeL by evaluating the experience of the affordances of the e-learning, and aiming to predict the efficiency and effectiveness. For further reading about this survey, we refer to the above-mentioned paper.”
